# Development and validation of a novel scale to evaluate dog owners' knowledge and awareness of ticks and tick-borne diseases

**DOI:** 10.1016/j.onehlt.2026.101615

**Published:** 2026-10-17

**Authors:** Salar Zarrabi Ahrabi, Zeynep Dilşah Karaçam Yilmaz, Gökçe Merey, Sait Alizade, Sude Su, Irem Vatansever, Gürkan Akyildiz, Ayşen Gargili Keleş

**Affiliations:** a Department of Basic Health Science, Health Sciences Faculty, Marmara University, Istanbul, Türkiye; b Department of Midwifery, Health Sciences Faculty, Marmara University, Istanbul, Türkiye; c Graduate in Veterinary Internal Medicine, Department of Internal Medicine, Islamic Azad University, Science and Research Branch, Tehran, Iran; d Department of Basic Health Science, Health Sciences Institute, Marmara University, Istanbul, Türkiye; e Department of Veterinary Microbiology, Institute of Health Sciences, Balıkesir University, Türkiye

**Keywords:** Ticks, Tick-borne disease, Scale, Awareness

## Abstract

Ticks and tick-borne diseases represent a serious public health concern, posing significant risks to both human and animal health. Dogs can host a wide range of tick species, many of which serve as vectors for zoonotic pathogens. In this methodological study, we generated a 23-item pool and evaluated it with eight experts to establish content validity. After a preliminary pilot test to ensure item clarity, the draft scale was administered to 100 dog owners visiting a veterinary clinic in Istanbul. Content validity was assessed using Lawshe's method, construct validity was examined through Exploratory and Confirmatory Factor Analyses, and reliability was evaluated using item–total correlations and Cronbach's alpha. All statistical analyses were performed using SPSS 27.0 and AMOS 24.0, with *p* < .05 considered significant. Item–total correlation analysis resulted in the removal of four items with coefficients below 0.30, while the remaining items showed acceptable values ranging from 0.381 to 0.634. After refinement based on reliability and factor-analytic criteria, the final 17-item scale demonstrated strong internal consistency (Cronbach's alpha = 0.88). Descriptive analyses indicated that the lowest awareness levels were associated with personal protective measures against tick exposure.

## Highlights

• A 17-item scale was developed to assess dog owners' awareness of ticks and tick-borne diseases.
• EFA and CFA confirmed a four-factor structure explaining 62.42% of the variance.
• The scale demonstrated high internal consistency (Cronbach's α = 0.88).
• Dog owners showed low awareness of tick removal and personal protective measures.
• The scale showed acceptable model fit (RMSEA = 0.071; CFI = 0.920).

## 1 Introduction

Ticks (Acari: *Ixodidae*) are hematophagous arthropods that play a significant role in the transmission of pathogens, including viruses, bacteria, and protozoa [1], [2], [3]. Their ability to affect both human and animal populations creates both public and veterinary health concerns because the incidence and geographic distribution of tick-borne diseases (TBDs) such as Lyme disease, anaplasmosis, ehrlichiosis, babesiosis, and Crimean-Congo Hemorrhagic Fever are increasing globally, driven by several factors, including climate change, which affects tick survival, reproduction, and distribution [4], [5]. Climate change and altered precipitation patterns have facilitated the expansion of tick habitats into previously non-endemic areas, thereby increasing human exposure to tick vectors. Additionally, changes in land use, such as reforestation and suburban development, have led to increased human-tick interactions [6], [7], [8]. Although mosquitoes are the most common vectors in the transmission of vector-borne diseases globally [9], in certain regions, such as the USA and Europe, ticks are the primary vectors, often associated with more severe and potentially fatal diseases, highlighting their critical importance in public health. Thus, they are recognized as very important vectors of human and animal pathogens, surpassing mosquitoes in temperate regions, and are responsible for transmitting a diverse array of infectious agents [10], [11]. Several studies have shown that ticks account for more than 95% of vector-borne disease cases in the United States and 50–70% in Europe. For instance, Lyme disease, caused by the spirochete *Borrelia burgdorferi* and primarily transmitted by Ixodes ticks, is the most prevalent tick-borne disease in the Northern Hemisphere, with an estimated annual incidence exceeding 476,000 cases [12].

The most important strategies for combating ticks and TBDs include personal protective measures, environmental control practices, and veterinary measures to protect animal health. Therefore, understanding the biology and ecology of ticks is crucial for disease prevention and control [13], [14]. In recent years, awareness of ticks and tick-borne diseases has gained increasing attention worldwide, as evidenced by a growing body of research conducted in diverse geographical settings. For instance, a 2017 study on tick-borne encephalitis and Lyme disease highlighted significant gaps in public understanding and emphasized the need for targeted educational interventions [15]. Similarly, a 2019 investigation in endemic regions revealed that 83% of participants reported that they were regularly checking their pets for ticks, indicating a relatively high level of preventive behavior among pet owners [16], while in eastern Bhutan, where agriculture and livestock farming are common livelihoods, 52% of individuals engaged in these sectors were found to be aware of tick-borne diseases, suggesting that occupational exposure may contribute to greater awareness [17]. In the United States, a study among veterinarians in Illinois demonstrated that those who had received training on tick-borne diseases within the past five years possessed significantly higher levels of awareness and knowledge on diagnostics [18]. Furthermore, an awareness study conducted in the Upper Midwest region of the USA in 2022 measured public knowledge regarding Lyme disease [19].

Despite these valuable contributions, there remains a notable gap in the literature regarding the specific awareness levels of dog owners, who constitute a key risk group due to their close contact with companion animals that may act as frequent hosts for multiple tick species. Especially, dogs are at risk of acquiring and potentially disseminating TBDs, some of which are zoonotic and pose risks to human health [20], [21]. Canine tick-borne diseases are of increasing concern in veterinary medicine due to their wide distribution and clinical severity [6], [22], [23]. Especially for Lyme disease, dogs play a critical role as both sentinels and reservoirs in Lyme ecology; studies have shown that domestic dogs frequently harbor *Ixodes ricinus* and *Ixodes scapularis*, which are competent vectors of *B. burgdorferi*, thereby increasing the risk of zoonotic transmission in peri-urban and rural areas [24], [25]. In a study conducted in the UK, the prevalence of *B. burgdorferi* in 4750 ticks collected from dogs was reported as 2% [26]. Moreover, in some regions, the prevalence of TBDs in dogs correlates with local human case rates, underlining the importance of canine surveillance in public health strategies [27], [28].

From a One Health perspective, integrated tick surveillance in both human and animal populations is critical for risk mapping and early intervention. Nevertheless, raising awareness of tick-borne diseases in Türkiye remains critically important from a public health perspective, as CCHF remains a major concern in endemic regions due to its high case fatality rate, which may reach up to 30% [29], [30], [31], [32], and over 17,000 confirmed human cases were reported between 2002 and 2024 [33], largely linked to *Hyalomma* tick exposure in rural areas and among agricultural workers [34], [35], [36]. To date, only a single study has reported evidence of 18% seropositivity for CCHF virus in 11 examined dogs [37], but still, in this context, the inclusion of companion animals in surveillance and prevention strategies could enhance early warning systems and mitigate transmission risks in endemic areas.

According to a 2024 report by FEDIAF (Federation of European Pet Food Industries), approximately 1.409 million dogs are owned in Türkiye [38], while several million more live as strays [39], adding complexity to the epidemiology of tick-borne diseases (TBDs) in the country. Therefore, increasing awareness among dog owners about ticks and TBDs is crucial, given their potential role in disease transmission and prevention.

This study aims to develop and validate a psychometric scale that evaluates dog owners' awareness, knowledge, and preventive behaviors regarding ticks and tick-borne diseases (TBDs), with particular emphasis on Lyme disease, the most prevalent TBD in both North America and Europe. Given the increasing incidence and geographic expansion of Lyme disease, as well as other zoonotic threats posed by ticks, the scale is designed to understand the public awareness of ticks and TBDs among dog owners. By addressing these gaps, the findings of this study aim to contribute to One Health strategies that integrate human, animal, and environmental health in the control and prevention of vector-borne diseases.

## 2 Methods

### 2.1 Research design

This study employed a methodological scale development design to develop and validate a measurement tool assessing dog owners' awareness of ticks and tick-borne diseases. The procedures described by DeVellis and Thorpe [40] were systematically followed. Accordingly, the scale development process consisted of six sequential stages: defining the construct through an extensive literature review; determining the scale format and generating an item pool; evaluating the items based on expert opinion; conducting a pilot study to assess clarity and comprehensibility; administering the draft scale to the target sample; and performing psychometric analyses to finalize the scale.

### 2.2 Scale development

There is no existing scale to measure awareness of ticks and tick-borne diseases among domestic dog owners. Therefore, the Tick and Tick-Borne Diseases Awareness Scale for Dog Owners was developed following standard scale development procedures [40], including literature review, item pool creation, expert evaluation, preliminary testing for clarity, administration to the sample group, and final data analysis.

First, studies on ticks, tick-borne diseases, and interactions among dogs, humans, and ticks were reviewed, with a focus on zoonotic diseases relevant to public health. Key statements reflecting the study's core concepts were drafted, forming a 23-item pool. These items were evaluated by eight experts *(four veterinarians, two public health specialists, and two researchers experienced in tick-borne diseases*), resulting in a 23-item draft scale. Preliminary testing with 15 individuals confirmed comprehensibility, after which the scale was administered to the sample group and analyzed to produce the final version. This final scale was developed and administered in Turkish, as all participants were native Turkish speakers. Therefore, no translation or cultural adaptation process was necessary.

### 2.3 Participant-reported outcomes questionnaires

Data were collected face-to-face using a Personal Information Form and the Tick and Tick-Borne Diseases Awareness Scale. Personal Information Form: The form was developed based on the literature [41] and included eight questions regarding participants' age, gender, educational level, employment status, occupation, duration of dog ownership, the dog's living environment, and regular veterinary care.

Tick and Tick-Borne Diseases Awareness Scale: The scale items were generated through a literature review [16], [41]. The initial version of the scale consisted of 23 items and was designed as a 5-point Likert scale (Absolutely Disagree, Disagree, Undecided, Agree, Strongly Agree).

### 2.4 Participants

Although various methods exist in the literature for determining sample size in scale development studies, it is generally recommended that the number of participants be five to ten times the number of items in the scale [42], [43]. Accordingly, considering the 23 items in the initial scale, the minimum target sample size was calculated as 115 participants. However, due to practical constraints, the study was completed with 100 participants. In addition, a post hoc power analysis was conducted using G*Power (version 3.1.9.6) to evaluate the adequacy of the sample size. The analysis, based on a medium effect size (d = 0.28), α = 0.05, and power of 80% (1–β = 0.80), indicated that the sample size was sufficient for preliminary scale development. Therefore, the final sample size was considered acceptable based on both methodological recommendations and power analysis.

### 2.5 Inclusion criteria

Being the owner of a domestic dog, visiting the selected veterinary clinic during the study period, and being willing to provide informed consent to participate in the study.

### 2.6 Validity

In this study, the validity of the developed scale was assessed through content and construct validity analyses. Content validity, which evaluates whether each item adequately represents the construct, was determined using the three-point method proposed by [44]. Eight experts rated each item, and the Content Validity Index (CVI) was calculated, with a recommended minimum value of 0.78 for eight experts [45].

Construct validity was evaluated by examining the underlying factor structure through Exploratory Factor Analysis (EFA) and subsequently testing the model using Confirmatory Factor Analysis (CFA) on the same dataset of 100 participants. Model fit in CFA was assessed using multiple goodness-of-fit indices. When the initial model did not demonstrate acceptable fit, model re-specification was conducted based on modification indices (MI) obtained from the analysis. Error covariances between item residuals were added when supported by high MI values and when theoretically justifiable. These modifications were implemented in a stepwise and theory-driven manner to improve model fit while preserving the conceptual integrity of this scale.

### 2.7 Reliability

Reliability of the scale was assessed using item-total correlation and internal consistency. Higher item-total correlations indicate stronger relationships between items and the construct being measured [46]. Internal consistency was evaluated using Cronbach's Alpha, which determines whether all items measure the same construct homogeneously [43]. A Cronbach's Alpha value of ≥0.60 is recommended for scale development studies.

### 2.8 Data analysis

Statistical analyses were performed using SPSS 27.0 and AMOS 24.0. No missing data were observed. The reliability of the scale was assessed using Pearson's test-retest method for stability, while item-total correlations and Cronbach's alpha were used to evaluate internal consistency. Content validity was assessed using Lawshe's technique, and construct validity was examined through Exploratory Factor Analysis (EFA) and Confirmatory Factor Analysis (CFA). In all analyses, *p* < .05 was considered statistically significant.

### 2.9 Ethics statement

Ethical approval was obtained from the Non-Interventional Clinical Research Ethics Committee of Marmara University Faculty of Health Sciences (22.05.2025/89). The study was conducted in accordance with the principles of the Declaration of Helsinki. All participants were informed about the study and their rights and provided written informed consent.

## 3 Results

### 3.1 Findings related to descriptive information

The mean age of the participants was 39.37 ± 12.92 years, and they had owned a dog for an average of 7.10 ± 6.82 years. Most participants held a bachelor's degree (54%, *n* = 54) and were employed (69%, *n* = 69). Additionally, 91% (*n* = 91) of participants reported that their dogs were kept at home and received regular veterinary care.

Analysis of the scores on the scale items revealed that the highest scores were obtained for Item 13 (*regular external parasite treatment*) and Item 19 (*awareness of serious human diseases caused by tick bites*). The lowest scores were observed for Item 23 (*personal protective measures against ticks*) and Item 11 (*knowledge of proper tick removal from the dog*) (Table 1).

**Table 1:** Descriptive statistics of item scores in the Tick and Tick-Borne Diseases Awareness Scale (*n* = 100).

Items	Strongly disagree n (%)	Disagree n (%)	Undecided n (%)	Agree n (%)	Strongly agree n (%)	Mean	SD
**Item 1.** I take my dog for regular daily walks.	3 (3.0%)	5 (5.0%)	3 (3.0%)	21 (21.0%)	68 (68.0%)	4.46	0.98
**Item 2.** I walk my dog in parks or green recreational areas.	6 (6.0%)	3 (3.0%)	5 (5.0%)	25 (25.0%)	61 (61.0%)	4.32	1.10
**Item 3.** Do you take your dog for walks in parks or green areas?	⁎						
**Item 4.** I am aware of areas in my region where ticks are commonly found.	16 (16.0%)	12 (12.0%)	24 (24.0%)	20 (20.0%)	28 (28.0%)	3.32	1.41
**Item 5.** I believe that the areas where I walk my dog may also pose a tick risk for myself and my family	15 (15.0%)	10 (10.0%)	16 (16.0%)	17 (17.0%)	42 (42.0%)	3.61	1.48
**Item 6.** I inspect my dog's body for ticks after returning from outdoor activities.	10 (10.0%)	16 (16.0%)	15 (15.0%)	24 (24.0%)	35 (35.0%)	3.58	1.37
**Item 7.** I am able to recognize a tick attached to my dog	4 (4.0%)	6 (6.0%)	15 (15.0%)	25 (25.0%)	50 (50.0%)	4.11	1.11
**Item 8.** Do you regularly use ectoparasite prevention products (e.g., tick collars) for your dog?	⁎⁎						
**Item 9.** Do you get informed by your veterinarian about tick-borne diseases?	⁎						
**Item 10.** Are you aware that ticks can act as vectors for diseases in dogs?	⁎						
**Item 11.** I know how to properly remove a tick attached to my dog.	24 (24.0%)	8 (8.0%)	21 (21.0%)	17 (17.0%)	30 (30.0%)	3.21	1.54
**Item 12.** I regularly use ecto parasite preventive products (e.g., tick collars) for my dog.	14 (14.0%)	3 (3.0%)	8 (8.0%)	16 (16.0%)	59 (59.0%)	4.03	1.43
**Item 13.** I ensure that my dog receives routine ecto parasite treatments	3 (3.0%)	1 (1.0%)	2 (2.0%)	16 (16.0%)	78 (78.0%)	4.65	0.83
**Item 14.** I obtain information about tick-borne diseases from my veterinarian	6 (6.0%)	3 (3.0%)	15 (15.0%)	31 (31.0%)	45 (45.0%)	4.06	1.12
**Item 15.** Are you aware that your dog should be vaccinated against Lyme disease?	⁎⁎						
**Item 16.** I am aware that Lyme disease is one of the illnesses that ticks can transmit to both humans and dogs.	18 (18.0%)	5 (5.0%)	12 (12.0%)	14 (14.0%)	51 (51.0%)	3.75	1.55
**Item 17.** I know that my dog should be vaccinated against Lyme disease.	13 (13.0%)	2 (2.0%)	11 (11.0%)	25 (25.0%)	49 (49.0%)	3.95	1.36
**Item 18.** I ensure that my dog receives the Lyme vaccination regularly.	24 (24.0%)	6 (6.0%)	19 (19.0%)	10 (10.0%)	41 (41.0%)	3.38	1.62
**Item 19.** I am aware that tick bites can cause serious diseases in humans.	3 (3.0%)	1 (1.0%)	4 (4.0%)	26 (26.0%)	66 (66.0%)	4.51	0.87
**Item 20.** Are you aware that Lyme disease is among the illnesses that ticks can transmit to humans and dogs?	⁎						
**Item 21.** When I find a tick on my dog, I also check my body for ticks.	5 (5.0%)	5 (5.0%)	4 (4.0%)	23 (23.0%)	63 (63.0%)	4.34	1.10
**Item 22**. I am knowledgeable about the potential health risks associated with tick bites and the evidence-based actions that should be undertaken in response.	4 (4.0%)	3 (3.0%)	22 (22.0%)	30 (30.0%)	41 (41.0%)	4.01	1.05
**Item 23**. I take protective measures against ticks, such as wearing long-sleeved and long-legged clothing, performing body checks, and using insect repellent.	23 (23.0%)	14 (14.0%)	20 (20.0%)	20 (20.0%)	23 (23.0%)	3.06	1.48

Min = Minimum, Max = Maximum, SD=Standard Deviation

⁎ Items were removed based on the item–total correlation analysis.

⁎⁎ Items were removed following the exploratory factor analysis (EFA).

Considering that the maximum possible score on the developed scale is 85, the participants in this study had a total mean score of 66.35 ± 12.45 on the Tick-Borne Diseases Awareness Scale of dog owners.

### 3.2 Content validity

To determine the content validity of the scale, an evaluation form consisting of the scale items was sent to eight experts, and their opinions were obtained. Based on the expert feedback, no items were removed, and the Content Validity Index (CVI) for the 23 items was calculated as 0.87. Accordingly, the content validity of the scale items was established.

### 3.3 Findings related to item-total correlation and internal consistency reliability of the tick and tick-borne diseases awareness scale of dog owners

Analysis of the item-total correlation reliability of the Tick and Tick-Borne Diseases Awareness Scale of Dog Owners revealed the correlation coefficients between each item and the total scale score. Items with coefficients below 0.30 were removed. Accordingly, Items 3 (*visiting natural/wooded areas with the dog*), 9 (*history of tick exposure in the dog*), 10 (*recurrent tick exposure in the dog*), and 20 (*perceived personal risk from ticks on the dog*) from the draft scale were excluded due to correlation coefficients below 0.30. The item-total correlation coefficients were then recalculated for the remaining items, and all were found to exceed 0.30. The coefficients of the remaining items ranged from 0.381 to 0.634, indicating that the items were appropriately interrelated. (Table 2).

**Table 2:** Item-total correlation coefficients of the Tick and Tick-Borne Diseases Awareness Scale for Dog Owners (*n* = 100).

Items	Initial item-total score correlation coefficients		Initial cronbach alfa	Final item-total score correlation coefficients		Final cronbach alfa
	r	p	α	r	p	α
			0.870		*p* < .0.001	0.887
Item 1	0.413	p < .0.001		0.401	p < .0.001	
Item 2	0.433	p < .0.001		0.447	p < .0.001	
Item 3	0.252	p < .0.001		Correlation below 0.30⁎		
Item 4	0.566	p < .0.001		0.577	p < .0.001	
Item 5	0.464	p < .0.001		0.470		
Item 6	0.528	p < .0.001		0.559		
Item 7	0.618	p < .0.001		0.634		
Item 8	0.554	p < .0.001		0.578	Cross-loaded on two factors⁎⁎⁎⁎⁎	
Item 9	0.238	p < .0.001		Correlation below 0.30⁎		
Item 10	0.090	p < .0.001		Correlation below 0.30⁎		
Item 11	0.411	p < .0.001		0.381		
Item 12	0.583	p < .0.001		0.608		
Item 13	0.428	p < .0.001		0.500		
Item 14	0.600	p < .0.001		0.601		
Item 15	0.608	p < .0.001		0.603	Cross-loaded on two factors⁎⁎	
Item 16	0.615	p < .0.001		0.590		
Item 17	0.571	p < .0.001		0.568		
Item 18	0.405	p < .0.001		0.417		
Item 19	0.496	p < .0.001		0.545		
Item 20	0.133	p < .0.001		Correlation below 0.30⁎		
Item 21	0.336	p < .0.001		0.362		
Item 22	0.614	p < .0.001		0.610		
Item 23	0.461	p < .0.001		0.418		

⁎ Items with item total score correlation values below 0.30 were removed.

⁎⁎ Items with cross-loadings factor loadings during EFA were removed.

⁎⁎⁎ Items with CFA values below 0.70 were removed.

To assess the internal consistency of the scale, Cronbach's Alpha coefficient was calculated. A higher Cronbach's Alpha indicates that the items in the scale are more consistent with each other and measure the same construct [47]. Since the coefficient ranges from 0 to 1, a higher value suggests that the items form a homogeneous structure. The draft version of the Tick and Tick-Borne Diseases Awareness Scale, consisting of 23 items, had a Cronbach's Alpha of α = 0.87. Following further analyses, the final scale included 17 items with an overall α = 0.88 (Table 3).

**Table 3:** Internal consistency reliability (Cronbach's alpha) of the total scale and sub-dimensions.

	Item no	α
Factor 1	4,5,16,17,18	0.82
Factor 2	6,12,13,19,21,23	0.77
Factor 3	7,11,14,22	0.76
Factor 4	1,2	0.82
Total	1,2,4,5,6,7,11,12,13,14,16,17,18,19,21,22,23	0.88

α = Cronbach Alpha.

The internal consistency of the four sub-dimensions identified through factor analyses was also evaluated. Cronbach's Alpha coefficients were found to be α = 0.82 for the first sub-dimension, α = 0.77 for the second, α = 0.76 for the third, and α = 0.82 for the fourth (Table 3).

### 3.4 Construct validity

#### 3.4.1 Exploratory factor analysis (EFA)

Construct validity was assessed for the 19-item version of the scale. For construct validity to be established, the dataset must be suitable for factor analysis. Accordingly, the Kaiser–Meyer–Olkin (KMO) test and Bartlett's Test of Sphericity were used to determine the suitability of the dataset prior to conducting the Exploratory Factor Analysis (EFA). The KMO coefficient was found to be 0.794, and the chi-square value calculated for Bartlett's test was significant (χ^2^ = 935.872; df = 171; *p* < .001). These findings indicate that the dataset was appropriate and of adequate size for factor analysis.

The Maximum Likelihood method and Varimax Rotation were employed in the factor analysis, and the factor loadings of the 19-item scale were calculated. Items 8 and 15 were removed due to cross-loading on multiple factors. As a result, the final version of the scale comprised 17 items. To verify the findings, all analyses were repeated, and the EFA was conducted again for the 17-item scale. Accordingly, the Tick-Borne Diseases Awareness Scale of Dog Owners reached a four-factor structure with eigenvalues greater than 1.00, explaining 62.42% of the total variance (Table 4). Scree plots shown this factor structure is presented in Fig. 1.

1. Sub-Dimension: This factor consisted of five items (Items 4, 5, 16, 17, and 18). The factor loadings were 0.658, 0.678, 0.658, 0.872, and 0.807, respectively. The explained variance for this sub-dimension was 17.876%.
2. Sub-Dimension: This factor consisted of six items (Items 6, 12, 13, 19, 21, and 23). The factor loadings were 0.526, 0.632, 0.754, 0.797, 0.648, and 0.508, respectively. The explained variance for this sub-dimension was 17.633%.
3. Sub-Dimension: This factor consisted of four items (Items 7, 11, 14, and 22). The factor loadings were 0.675, 0789, 0.668, and 0.596, respectively. The explained variance for this sub-dimension was 15.674%.
4. Sub-Dimension: This factor consisted of two items (Items 1 and 2) (Table 4). The factor loadings were 0.881 and 0.849, respectively. The explained variance for this sub-dimension was 11.237%.

**Table 4:** Goodness-of-fit indices for the confirmatory factor analysis model.

RMSEA	CFI	IFI	AGFI	CMIN	CMIN/df
0.071	0.920	0.923	0.805	163.601	1.501

RMSEA = Root Mean Square Error of Approximation, CFI=Comparative Fit Index, NFI=Non-Normed Fit Index, GFI = Goodness-of-Fit Index, AGFI = Adjusted Goodness-of Fit Index, IFI=Incremental Fit Index.

**Fig. 1:**
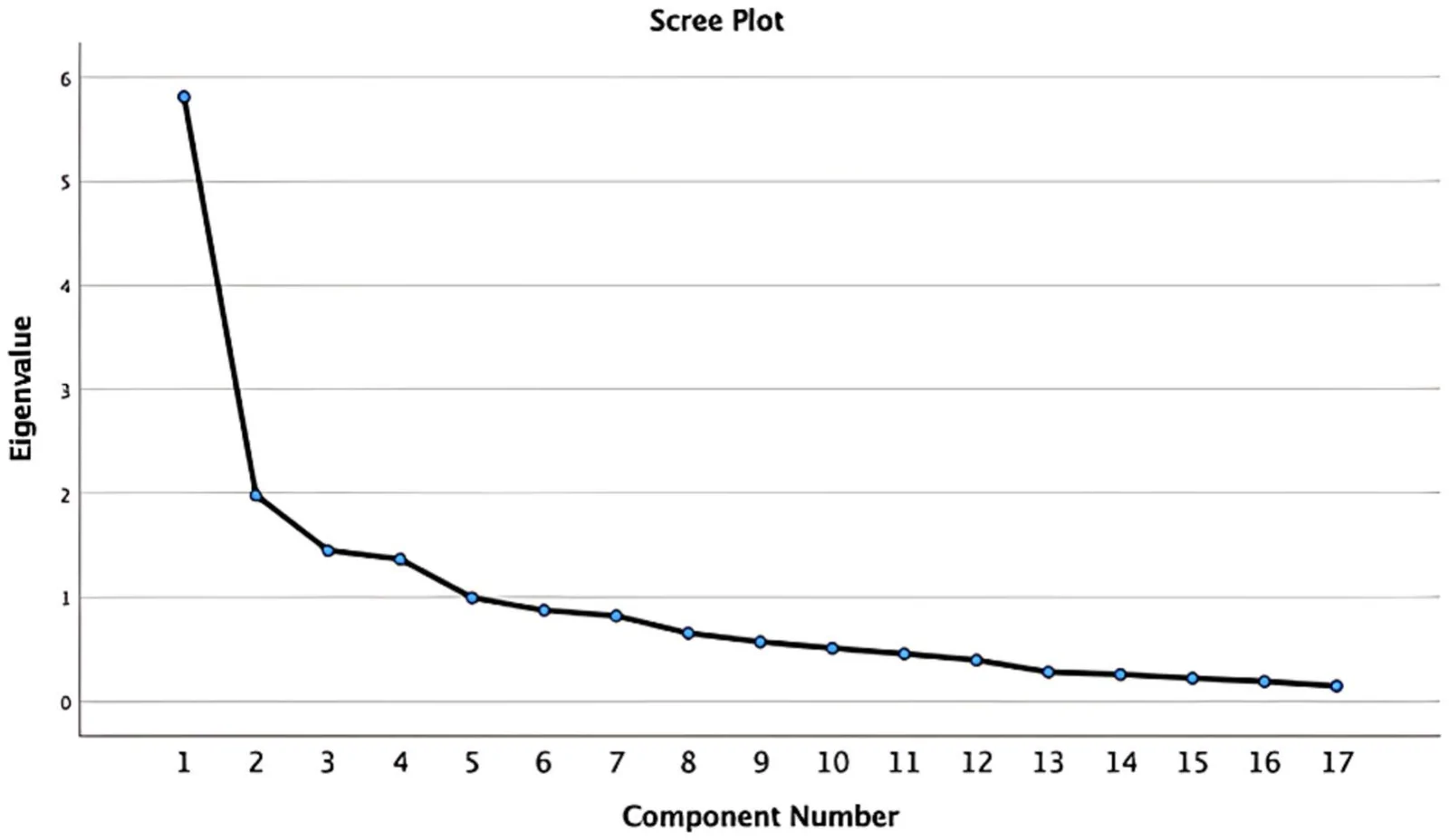
As a result of Exploratory Factor Analysis (EFA), factors with eigenvalues greater than 1 were accepted as significant and sub-dimensions were formed by considering the Scree Plot. In this context, it was determined that the Scree Plot in Fig. 1 showed a 4-factor structure.

#### 3.4.2 Confirmatory factor analysis (CFA)

According to the CFA, the Structural Equation Modeling results were significant at the *p* < .001 level, indicating that the 17 items and four sub-dimensions were appropriately related to the overall scale structure. The CFA results confirmed the four sub-dimension structure of the scale. This model is presented in Fig. 2.

**Fig. 2:**
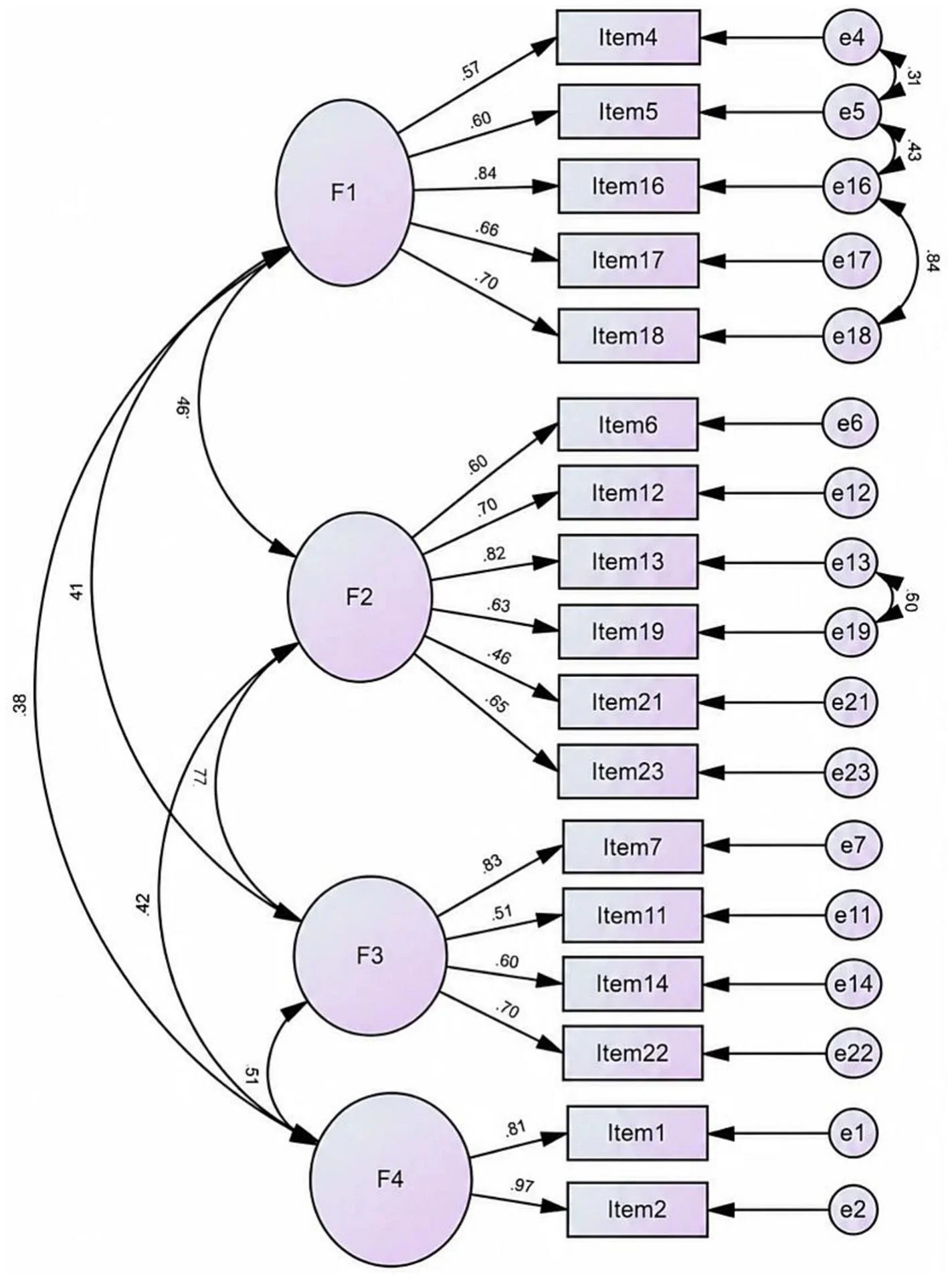
Path diagram of the Confirmatory Factor Analysis (CFA) model for the Tick and Tick-Borne Diseases Awareness Scale. The model consists of four sub-dimensions and 17 items, with standardized factor loadings shown on each path. To show contents of items, go to Table 1.

Model modifications were performed, during which variables that reduced model fit were identified, and new covariances were added between error terms with high covariance (e4-e5, e5-e16, e16-e18, and e13-e19). Following these modifications, the recalculated fit indices met the accepted criteria, as shown in the table below. Values above 0.90 for the GFI, IFI, and CFI indices, above 0.80 for the AGFI index, and below 0.080 for the RMSEA value indicate an acceptable model fit (Marsh et al., 2006). According to the first-level multifactor analysis results, the goodness-of-fit indices of the scale demonstrated acceptable fit, with RMSEA = 0.071, AGFI = 0.805, CFI = 0.920, and χ^2^ = 1.501 (*p* < .001) (Table 4).

## 4 Discussion

In this study, the Tick and Tick-Borne Diseases Awareness Scale was developed to assess the awareness of dog owners regarding ticks and tick-borne diseases. The final scale consists of 17 items and four sub-dimensions, and its psychometric evaluation demonstrated that it is a valid and reliable measurement tool.

The findings indicate that dog owners possess a basic awareness of ticks and tick-borne diseases. The majority of participants reported consistently engaging in behaviors such as taking their dogs for regular walks and providing routine parasite preventive treatments. This finding aligns with previous studies in the literature, which have reported that dog owners generally demonstrate a high level of awareness regarding protective behaviors [48], [49], [50].

On the other hand, awareness regarding Lyme disease, particularly in relation to vaccination, was relatively low. The mean score for item 18, which assesses regular Lyme vaccination practices, was lower compared to other items, suggesting that dog owners may have insufficient knowledge about vaccination practices and the significance of Lyme disease. Similarly, a recent study conducted in France also reported low awareness regarding the Lyme vaccine [51]. Similarly, the low scores and high standard deviations observed for personal protective measures indicate heterogeneity in knowledge and practices among individuals. This finding is consistent with the literature, which reports gaps in public health awareness and the adoption of personal protective measures [16].

Although participants demonstrated a high level of awareness regarding protective behaviors for their dogs, it is noteworthy that their awareness of personal protective behaviors was comparatively lower. This suggests that while dog owners tend to perceive tick-borne diseases primarily as a threat to their pets, they may not consistently translate this awareness into behaviors that mitigate the risk for themselves. Furthermore, the moderate scores observed for items related to knowledge of Lyme disease and other tick-borne illnesses indicate that knowledge alone does not necessarily translate into protective behaviors. Collectively, these findings highlight the importance of awareness programs that address both animal health and personal protective behaviors against ticks and tick-borne diseases [52].

Item-total Correlation Analyses were conducted to assess the representativeness of each item in the developed scale. In this regard, it is recommended that item-total correlation values for Likert-type scales fall between 0.20 and 0.80, and that items with values below 0.20 or above 0.80 be removed from scale [43], [46], [53].

In this study, Items 3, 9, 10, and 20 were removed due to low item-total correlations. A closer examination of these items indicates that they do not adequately represent the construct of awareness targeted by the scale. Specifically, Items 9 and 10 assess past experiences rather than awareness, and prior exposure to ticks does not necessarily reflect an individual's level of awareness. Similarly, Item 3 reflects environmental exposure (e.g., visiting wooded or natural areas) rather than awareness itself. This item also overlaps conceptually with Items 1 and 2, which assess routine dog-walking behaviors, potentially reducing its discriminative contribution within the scale. Furthermore, Item 20 focuses on perceived personal risk rather than objective awareness. As such, it may be influenced by subjective interpretation and may not align consistently with the overall construct measured by the scale [43], [46].

To assess construct validity, Exploratory Factor Analysis (EFA) and Confirmatory Factor Analysis (CFA) were conducted. For EFA, a Kaiser–Meyer–Olkin (KMO) value of at least 0.70 is recommended to ensure the adequacy of the sample [54], [55], [56]. In this study, the 19-item version of the scale yielded a KMO value of 0.794, indicating sufficient sample adequacy. EFA results revealed a four-factor structure in the 19-item version of the scale. Items with cross-loadings were removed, as they may disrupt the factor structure and fail to adequately represent the intended construct. Accordingly, Items 8 and 15 were excluded, and the analyses were repeated with the remaining 17 items.

A closer examination of these items indicates that they represent general knowledge about ticks, such as their seasonal activity and their role in disease transmission. However, such knowledge may span multiple domains, including awareness, knowledge, and preventive behavior. This conceptual overlap may have led to ambiguity in participants' responses, causing these items to load on more than one factor. Their removal resulted in a clearer and more interpretable factor structure.

The final 17-item version of the Tick and Tick-Borne Diseases Awareness Scale explained 62.42% of the total variance, with each sub-dimension accounting for between 11.23% and 17.87% of the variance, indicating that each sub-dimension made a meaningful contribution consistent with the literature [43], [57].

When the sub-dimensions were examined conceptually, it was observed that the first sub-dimension represents risk awareness, the second reflects preventive behaviors, the third corresponds to practical management skills, and the fourth is related to exposure-related behaviors. This four-factor structure suggests that awareness of tick-borne diseases is not a unidimensional construct but consists of multiple interrelated components, including knowledge, behavior, and practical skills.

To confirm this structure, CFA was conducted, and the results indicated that the model demonstrated an acceptable fit. Accordingly, the final version of the scale consisted of 17 items and four sub-dimensions. Finally, this scale demonstrates high internal consistency (Cronbach's α = 0.88) [[55], [58]].

Tick-borne diseases represent a critical category of vector-borne infections with significant implications for both human and animal health. The findings of this study reveal notable gaps in awareness among the general public, pet owners, and veterinary professionals. In Turkey, particularly in Istanbul, the combination of high human population density, the presence of domestic and stray animals, and environments conducive to certain tick species underscores the necessity of targeted education. Enhancing knowledge on tick ecology, promoting the regular use of parasite prevention strategies for pets, and ensuring timely veterinary consultation are essential measures to mitigate the risk of disease transmission.

## 5 Conclusion

The findings highlight that veterinarians and public health professionals should not only teach dog owners basic protective behaviors but also raise awareness about more specific issues, such as Lyme disease, vaccination, and personal protective measures. Furthermore, the observed high standard deviations indicate that educational programs should account for individual differences and be more targeted and participant centered. In conclusion, although dog owners generally demonstrate a high level of awareness, gaps remain in certain knowledge and practice areas, and addressing these gaps is crucial for both canine and public health.

## 6 Limitations

This study has several limitations. One important limitation is that both exploratory and confirmatory factor analyses were conducted on the same dataset. Although this approach is commonly used in preliminary scale development studies with relatively small sample sizes, it may increase the risk of overfitting and limit the generalizability and robustness of the factor structure. Therefore, future studies are recommended to validate the scale using an independent sample or by employing split-sample validation techniques. Another limitation of this study is that the sample was drawn from a single urban setting in Istanbul. Therefore, these findings may not be fully generalizable to rural populations, where environmental exposure to ticks, lifestyle factors, and access to veterinary services may differ significantly. Future studies are recommended to validate this scale in rural populations and across diverse geographical regions to enhance its generalizability.

## CRediT authorship contribution statement

**Salar Zarrabi Ahrabi:** Writing – review & editing, Writing – original draft, Supervision, Project administration, Methodology, Conceptualization. **Zeynep Dilşah Karaçam Yilmaz:** Writing – review & editing, Software, Formal analysis. **Gökçe Merey:** Writing – review & editing, Writing – original draft, Conceptualization. **Sait Alizade:** Investigation, Data curation. **Sude Su:** Investigation, Data curation. **Irem Vatansever:** Investigation, Data curation. **Gürkan Akyildiz:** Writing – review & editing, Writing – original draft, Methodology, Conceptualization. **Ayşen Gargili Keleş:** Writing – review & editing, Writing – original draft, Supervision, Project administration, Methodology, Conceptualization.

## Ethics declaration

Written informed consent to take part in the study and to publish the article has been obtained from all participants or their legal representatives. The privacy rights of participants have been observed.

This study was performed in compliance with relevant laws, regulatory frameworks and guidelines where the research took place. This study was approved by the Marmara university. (Approval No. 22.05.2025/89).

## Funding

The study received no external funding.

## Declaration of competing interest

None of the authors has a conflict of interest concerning this manuscript.

## Data Availability

Data will be made available on request.

## References

[1] Estrada-Peña A., de la Fuente J. (2014). The ecology of ticks and epidemiology of tick-borne viral diseases. Antivir. Res..

[2] Perveen N., Muhammad K., Muzaffar S.B., Zaheer T., Munawar N., Gajic B., Sparagano O.A., Kishore U., Willingham A.L. (2023). Host-pathogen interaction in arthropod vectors: lessons from viral infections. Front. Immunol..

[3] Ali A., Mulenga A., Vaz I.S. (2020). Tick and tick-borne pathogens: molecular and immune targets for control strategies. Front. Media SA.

[4] Kar S., Keles A.G. (2021). Climate, Ticks and Disease.

[5] Eleftheriou A., Swisher S., Arruda A., Berrian A., Pesapane R. (2023). A survey of knowledge, attitudes, and practices of veterinary professionals regarding ticks and tick-borne diseases: insights from Ohio, USA. One Health..

[6] de la Fuente J., Estrada-Peña A., Rafael M., Almazán C., Bermúdez S., Abdelbaset A.E., Kasaija P.D., Kabi F., Akande F.A., Ajagbe D.O. (2023). Perception of ticks and tick-borne diseases worldwide. Pathogens.

[7] Ortiz D.I., Piche-Ovares M., Romero-Vega L.M., Wagman J., Troyo A. (2021). The impact of deforestation, urbanization, and changing land use patterns on the ecology of mosquito and tick-borne diseases in Central America. Insects.

[8] Diuk-Wasser M.A., VanAcker M.C., Fernandez M.P. (2021). Impact of land use changes and habitat fragmentation on the eco-epidemiology of tick-borne diseases. J. Med. Entomol..

[9] Parnell S.T., Green W.L., Naganathan S., Katz E., Velez L.I., Gajurel K. (2024). Globally connected and universally at risk: mosquito-borne diseases in the 21st century. Emerg. Med. Rep..

[10] Rochlin I., Toledo A. (2020). Emerging tick-borne pathogens of public health importance: a mini-review. J. Med. Microbiol..

[11] Estrada-Peña A., Venzal J.M., Kocan K.M., Sonenshine D.E. (2008).

[12] Kernif T., Leulmi H., Raoult D., Parola P. (2016). Emerging Infections.

[13] Belobo J.T.E., Kenmoe S., Kengne-Nde C., Emoh C.P.D., Bowo-Ngandji A., Tchatchouang S., Sowe Wobessi J.N., Mbongue Mikangue C.A., Tazokong H.R., Kingue Bebey S.R. (2021). Worldwide epidemiology of Crimean-Congo Hemorrhagic Fever Virus in humans, ticks and other animal species, a systematic review and meta-analysis. PLoS Negl. Trop. Dis..

[14] Sedda L., Morley D., Braks M., De Simone L., Benz D., Rogers D. (2014). Risk assessment of vector-borne diseases for public health governance. Public Health.

[15] Zöldi V., Turunen T., Lyytikäinen O., Sane J. (2017). Knowledge, attitudes, and practices regarding ticks and tick-borne diseases, Finland. Ticks Tick Borne Dis..

[16] Niesobecki S., Hansen A., Rutz H., Mehta S., Feldman K., Meek J., Niccolai L., Hook S., Hinckley A. (2019). Knowledge, attitudes, and behaviors regarding tick-borne disease prevention in endemic areas. Ticks Tick-Borne Dis..

[17] Namgyal J., Tenzin T., Checkley S., Lysyk T.J., Rinchen S., Gurung R.B., Dorjee S., Couloigner I., Cork S.C. (2021). A knowledge, attitudes, and practices study on ticks and tick-borne diseases in cattle among farmers in a selected area of eastern Bhutan. PLoS One.

[18] Crist S.D., Kopsco H., Miller A., Gronemeyer P., Mateus-Pinilla N., Smith R.L. (2022). Knowledge, attitudes, and practices of veterinary professionals towards ticks and tick-borne diseases in Illinois. One Health.

[19] Beck A., Bjork J., Biggerstaff B.J., Eisen L., Eisen R., Foster E., Signs K., Tsao J.I., Kough E., Peterson M. (2022). Knowledge, attitudes, and behaviors regarding tick-borne disease prevention in Lyme disease-endemic areas of the Upper Midwest, United States. Ticks Tick Borne Dis..

[20] Shaw S.E., Day M.J., Birtles R.J., Breitschwerdt E.B. (2001). Tick-borne infectious diseases of dogs. Trends Parasitol..

[21] Dantas-Torres F., Chomel B.B., Otranto D. (2012). Ticks and tick-borne diseases: a One Health perspective. Trends Parasitol..

[22] Johnson N., Phipps L.P., Hansford K.M., Folly A.J., Fooks A.R., Medlock J.M., Mansfield K.L. (2022). One Health approach to tick and tick-borne disease surveillance in the United Kingdom. Int. J. Environ. Res. Public Health.

[23] Irwin P.J. (2009). Canine babesiosis: from molecular taxonomy to control. Parasit. Vectors.

[24] Littman M.P., Goldstein R.E., Labato M.A., Lappin M.R., Moore G.E. (2006). ACVIM small animal consensus statement on Lyme disease in dogs: diagnosis, treatment, and prevention. J. Vet. Intern. Med..

[25] Morea A., Schino V., Bartolomeo N., Ravallese R., Sacino G., Ravallese R., Iatta R., Otranto D., Lovreglio P., Stufano A. (2025). Seroprevalence of tick-borne diseases in Europe in occupational settings: a systematic review and metanalysis. Public Health.

[26] Abdullah S., Helps C., Tasker S., Newbury H., Wall R. (2018). Prevalence and distribution of Borrelia and Babesia species in ticks feeding on dogs in the UK. Med. Vet. Entomol..

[27] Beugnet F., Marié J.-L. (2009). Emerging arthropod-borne diseases of companion animals in Europe. Vet. Parasitol..

[28] Mead P., Hook S., Niesobecki S., Ray J., Meek J., Delorey M., Prue C., Hinckley A. (2018). Risk factors for tick exposure in suburban settings in the Northeastern United States. Ticks Tick-Borne Dis..

[29] Ergönül Ö. (2006). Crimean-Congo haemorrhagic fever. Lancet Infect. Dis..

[30] Bente D.A., Forrester N.L., Watts D.M., McAuley A.J., Whitehouse C.A., Bray M. (2013). Crimean-Congo hemorrhagic fever: history, epidemiology, pathogenesis, clinical syndrome and genetic diversity. Antivir. Res..

[31] Spengler J.R., Bergeron É., Rollin P.E. (2016). Seroepidemiological studies of Crimean-Congo hemorrhagic fever virus in domestic and wild animals. PLoS Negl. Trop. Dis..

[32] Tavassoli M., Ahrabi S.Z., Akyildiz G., Keles A.G. (2024). Crimean-Congo hemorrhagic fever, transfer patterns, vectors and history in Iran and neighboring countries. Vet. Res. Forum.

[33] Şahan S., Topluoğlu S., Temel F., Coşgun Y., Öz E., Demirkol M.E., Birinci Ş. (2025). Evaluation of epidemiological characteristics of Crimean-Congo haemorrhagic fever patients reported to the National Surveillance System in Türkiye, 2011-2024. Acta Trop..

[34] Ahrabi S.Z., Pınarlık F., Akyıldız G., Kuşkucu M., Kar S., Ergönül Ö., Keleş A.G., T.V.-B.I.S. Group (2025). Human tick biting and tick-borne disease risk in Türkiye: systematic review. PLoS Negl. Trop. Dis..

[35] Leblebicioglu H., Ozaras R., Irmak H., Sencan I. (2016). Crimean-Congo hemorrhagic fever in Turkey: current status and future challenges. Antivir. Res..

[36] Yilmaz G.R., Buzgan T., Irmak H., Safran A., Uzun R., Cevik M.A., Torunoglu M.A. (2009). The epidemiology of Crimean-Congo hemorrhagic fever in Turkey, 2002–2007. Int. J. Infect. Dis..

[37] Mangombi J.B., Roqueplo C., Sambou M., Dahmani M., Mediannikov O., Comtet L., Davoust B. (2020). Seroprevalence of Crimean-Congo hemorrhagic fever in domesticated animals in Northwestern Senegal. Vector Borne Zoonotic Dis..

[38] Rowan A.N., Rowan K. (2024).

[39] Özen D., Böhning D., Gürcan İ.S. (2016). Estimation of stray dog and cat populations in metropolitan Ankara, Turkey. Turk. J. Vet. Anim. Sci..

[40] DeVellis R.F., Thorpe C.T. (2021).

[41] Boehm S., Fingerle V., Beyerlein A., Wildner M., Böhmer M.M. (2025). Knowledge, attitudes and behaviour towards ticks and tick-borne diseases—a survey among Lyme borreliosis cases in Bavaria in 2019. Ticks Tick-Borne Dis..

[42] Tavşancil E., Keser H. (2002). Development of a Likert type attitude scale towards internet usage. J. Educ. Sci. Pract..

[43] Gökdemir F., Yılmaz T. (2023). Processes of using, modifying, adapting and developing Likert type scales. J. ET Nurs..

[44] Lawshe C.H. (1975). A quantitative approach to content validity. Pers. Psychol..

[45] Yurdugül H. (2005).

[46] Çapık C., Gözüm S. (2018). Intercultural scale adaptation stages, language and culture adaptation: updated guideline. Florence Nightingale J. Nurs..

[47] Akgül A., Çevik O. (2005).

[48] Panchim P., Saengpradub P., Rakkijpradit S., Watananontchai A., Chansiripornchai P., Angkanaporn K. (2024). Survey of dog owners' and veterinarians' attitudes regarding the selection of flea and tick prevention products in Thailand. Vet. World.

[49] Boost M.V., Tung C.-y., Ip C.H.-K., Man J.F.-o., Hui T.W.-t., Leung C.F.-y., Mak M.Y.-w., Yuen Q., O’Donoghue M.M. (2017). Awareness of tick-borne disease and compliance with using tick preventive products of dog owners in Hong Kong. Prev. Vet. Med..

[50] Beck S., Schein E., Baldermann C., von Samson-Himmelstjerna G., Kohn B. (2013). Tick infestation and tick prophylaxis in dogs in the area of Berlin/Brandenburg—results of a questionnaire study. Berl. Munch. Tierarztl. Wochenschr..

[51] Philibert C., Ferrolho J., Gibson M.S., Ramalho de Sousa S., Vilhena H., Anastácio S. (2025). The challenge of Lyme borreliosis: knowledge, attitudes, and practices in France. Biology.

[52] Torres P., Pienaar E.F., Ritchie M.A., Gabriel M.W., Yabsley M.J. (2025). Knowledge, attitudes, and practices of Native Americans in northern California regarding ticks and tick-borne diseases. One Health.

[53] Streiner D.L., Norman G.R., Cairney J. (2024).

[54] Verma J.P., Abdel-Salam A.-S.G. (2019).

[55] Shrestha N. (2021). Factor analysis as a tool for survey analysis. Am. J. Appl. Math. Stat..

[56] Nkansah B.K. (2018). On the Kaiser-Meier-Olkin’s measure of sampling adequacy. Math. Theory Model..

[57] Hair J.F. (2009).

[58] Boateng G.O., Neilands T.B., Frongillo E.A., Melgar-Quiñonez H.R., Young S.L. (2018). Best practices for developing and validating scales for health, social, and behavioral research: a primer. Front. Public Health.

